# Everybody's Page

**Published:** 1888-02-04

**Authors:** 


### Everybody's Page.

It forms a certain excuse for the much-condemned wearing of earrings that in certain cases a ring in the ear appears to act as a seton, or counter-irritant in the progress of some eye complaints.

A very severe form of deafness is to be met with among artillerymen and others frequently subjected to loud and jarring sounds. It may be in a great measure prevented by keeping a plug of cotton-wool in the ears. Another precaution which should be adopted by those who fire heavy guns is keeping the mouth open, instead, as is usually taught, of closing it and pressing the teeth together.

One of the recent movements for the help of the workers in our cities which deserves great praise, is the establishment of homes for single women. The girls who work in our factories and shops often live in lodgings where the comfort is small in proportion to what is paid, and where they do not receive that care and friendly guidance which almost every girl requires. The average landlady has no time for such things, and neither knows nor cares how her lodger spends her leisure hours. Therefore, these homes—managed on really home-like principles—with a kindly matron in whom the girls can confide, and occasional recreative evenings to brighten their lives, promise to be a great success; and ought to be better known both by working-women themselves and by the public at large. At present they are few in number, but the success of those at present established should encourage the formation of others, especially as they can and ought to be made self-supporting.

Knife and scizzors grinding is one of the unhealthiest trades a man can take up. The average number of years a grinder can work is thirteen. The fine particles of steel getting into the lungs set up inflammation which culminates in an abscess. When this begins to discharge some relief is experienced, but the disease invariably terminates fatally, though the victim sometimes lives as long as ten years after being obliged to give up work. The average duration of life in the various branches of the grinding trade is said to be as follows: Dry grinders of forks, 29 years; razors, 31 years; scissors, 32 years; edge-tool and wool-shears, 32 years; spring knives, 35 years; files, 35 years; saws, 38 years; and sickles, 38 years.

Another unhealthy trade is the making of white-lead, which has hitherto been manufactured only by a very lengthy and offensive process. The pig-lead was suspended over pots of acetic acid and covered with manure till the combined influences corroded it, making it into carbonate of lead, *i.e.* white lead. This process takes three or four months. It is noteworthy that when the lead is disinterred, women are employed in picking it out of its malodorous environment, not only because they will work for smaller wages, but because men cannot bear the smell. In this, as in most things, woman is the stronger to endure. While doing this there is great danger to the worker, not only from inhaling particles of white lead, but from the risk of blood poisoning being caused by contact with the corroded metal. For this reason, even more than because of the greater speed he claims, the world owes a debt of gratitude to Mr. Henry Condy, well known as the patentee of Condy's fluid, for the method he has discovered of making white lead in twenty-four hours by a process as simple as it is rapid, and absolutely free from danger to the operative. Besides these advantages, the cost of production is much reduced by the new process, so that white lead, an article which is essential in the arts, will soon be much cheaper than it has hitherto been.

Beware of too much quinine! It will produce a congestion of the ear and irritation of the auditory nerve. The common habit of taking quinine for neuralgia and other ailments without consulting a doctor is altogether reprehensible, and may lead to very serious results. Many cases of deafness are produced by over doses and long-continued use of this drug.

It is not generally known that a material loosely woven is warmer than the same material closely woven. Wool or cotton when carded and spread out as wadding is warmer than the same material would be if spun into cloth. This depends on the fact that air is a bad conductor of heat, and the loose material, by confining air within its meshes, adds materially to its warming properties.

The more vigorous are a man's physical movements, the less need has he of ablutions for the maintenance of his health, partly because the continued friction of most parts of the skin, and the perspiration produced by work are sufficient in themselves to perform the mechanical task of cleansing. Thus experience teaches that to the labourer baths and ablutions are less necessary than to a man of a sedentary mode of life, and to the adult than to the infant, whose lungs discharge their office feebly.

A very remarkable case is mentioned in the New York *Tribune*. Jennie Clifton, a handsome girl of sixteen, living at Arkwright, N.Y., has never seen the world by daylight, through able by lamplight to sew and read as well as anybody. Up to the age of four or five years she was believed to be totally blind. Then her parents noticed that after the lamp was lighted she gave evidence of seeing, and gradually this power of sight grew upon her until the little one played with her toys by artificial light as clearly as other children do by daylight; and this curious limitation of vision still continues.

In the old days of "resurrectioning," but before the task of providing for the necessities of the dissecting-room was given over to Messrs. Burke and Hare, and their like, medical students were encouraged to share the work. In Aberdeen the Medico-Chirurgical Society used to insist that every student should take his part in this hazardous and disagreeable business in turn, or pay a fine of 10*s.* 6*d.* as a recompense to those who did. It was agreed to pay 12*s.* 6*d.* to each student who helped in a successful "lifting," and they further received "twelve pence extra to warm the insides of their jackets." Possibly this twelve pence was meant to be spent on flannel; but, on the whole, it is more probable that the "inside of the jacket" should be taken metaphorically, and that the extra shilling went towards comforting the inner man with what our ancestors euphemistically called "strong waters."

Many trades, like those of the tailor, the compositor, and the sempstress, are harmful to the sight in consequence of the sustained exertion of the ciliary muscles in effecting the accommodation of the eye for near objects, which they require. Others, like that of the washerwoman, are injurious in consequence of the alternate exposure of the eyes to cold and damp heat. Others, like those of fitters, riveters, hammermen, and smiths of all kinds, owing to the liability to accident and the effects of bright fires. Others, like farm labourers, harvestmen, and reapers generally, are exposed to cold and to the singularly dangerous results of a slight scratch of the surface of the eye with an awn of barley or a blade of grass—dangerous because they are usually disregarded, and the man continues to work under a hot sun and drinks largely, while he perhaps takes little food. Loss of the eye often resuts in these cases from what might have been originally cured by the instillation of a drop of sweet oil, and the application of a wet pad and bandage, with rest of the eye for a few hours.

